# Investigating the long-term public health and co-benefit impacts of an urban greenway intervention in the UK: a natural experiment evaluation – study protocol

**DOI:** 10.1136/bmjopen-2024-097530

**Published:** 2025-07-06

**Authors:** Ruth F Hunter, Claire Cleland, Ruoyu Wang, Ciaran O’Neill, Shay Mullineaux, Christopher Tate, Hüseyin Küçükali, Selin Akaraci, Niamh O’Kane, Leandro Garcia, Mike Clarke, Christopher R Cardwell, Sophie Jones, Aideen Maguire, Geraint Ellis, Brendan Murtagh, Anna Jurek-Loughrey, Dominic Bryan, John Barry, Jeremy Hilton, Mehdi Hafezi, Natalie Clewley, Frank Kee

**Affiliations:** 1 Centre for Public Health, Queen’s University Belfast, Belfast, UK; 2 Institute of Public Health and Wellbeing, University of Essex, Essex, UK; 3 Population, Policy and Practice Department, University College London, London, UK; 4 School of Natural and Built Environment, Queen’s University Belfast, Belfast, UK; 5 School of Electronics, Electrical Engineering and Computer Science, Queen’s University Belfast, Belfast, UK; 6 School of History, Anthropology, Philosophy and Politics, Queen’s University Belfast, Belfast, UK; 7 Complex Systems Governance Group, Faculty of Engineering and Applied Science, Cranfield University, Cranfield, UK

**Keywords:** PUBLIC HEALTH, EPIDEMIOLOGY, Primary Prevention

## Abstract

**Introduction:**

Urban green and blue space (UGBS) interventions, such as the development of an urban greenway, have the potential to provide public health benefits and multiple co-benefits in the realms of the environment, economy and society. This paper presents the protocol for a 5-year follow-up evaluation of the public health benefits and co-benefits of an urban greenway in Belfast, UK.

**Methods and analysis:**

The natural experiment evaluation uses a range of systems-oriented and mixed-method approaches. First, using group model building methods, we codeveloped a causal loop diagram with stakeholders to inform the evaluation framework. We will use other systems methods including viable systems modelling and soft systems methodology to understand the context of the system (ie, the intervention) and the stakeholders involved in the development, implementation and maintenance phases. The effectiveness evaluation includes a repeat cross-sectional household survey with a random sample of 1200 local residents (adults aged ≥16 years old) who live within 1 mile of the greenway. The survey is complemented with administrative data from the National Health Service. For the household survey, outcomes include physical activity, mental well-being, quality of life, social capital, perceptions of environment and biodiversity. From the administrative data, outcomes include prescription medications for a range of non-communicable diseases such as cardiovascular disease, type II diabetes mellitus, chronic respiratory and mental health conditions. We also investigate changes in infectious disease rates, including COVID-19, and maternal and child health outcomes such as birth weight and gestational diabetes. A range of economic evaluation methods, including a cost-effectiveness analysis and social return on investment (SROI), will be employed. Findings from the household survey and administrative data analysis will be further explored in focus groups with a subsample of those who complete the household survey and the local community to explore possible mechanistic pathways and other impacts beyond those measured. Process evaluation methods include intercept surveys and direct observation of the number and type of greenway visitors using the Systems for Observing Play and Recreation in Communities tool. Finally, we will use methods such as weight of evidence, simulation and group model building, each embedding participatory engagement with stakeholders to help us interpret, triangulate and synthesise the findings.

**Ethics and dissemination:**

To our knowledge, this is one of the first natural experiments with a 5-year follow-up evaluation of an UGBS intervention. The findings will help inform future policy and practice on UGBS interventions intended to bring a range of public health benefits and co-benefits. Ethics approval was obtained from the Medicine, Health and Life Sciences Research Ethics Committee prior to the commencement of the study. All participants in the household survey and focus group workshops will provide written informed consent before taking part in the study. Findings will be reported to (1) participants and stakeholders; (2) funding bodies supporting the research; (3) local, regional and national governments to inform policy; (4) presented at local, national and international conferences and (5) disseminated by peer-review publications.

**STRENGTHS AND LIMITATIONS OF THIS STUDY**

- Long-term 5-year follow-up investigation.
- Survey instruments based on validated tools and administrative data.
- We will use a variety of systems approaches along with other qualitative and quantitative methods.
- The evaluation will focus on outcomes such as the incidence of non-communicable diseases (NCDs) and NCD risk factors, and other environmental, social and economic outcomes, as well as the impact on health inequalities.
- We incorporate a range of methods and comparator strategies in our analyses to reduce the risk of bias in the study.

## Introduction

 Many systematic reviews and meta-analyses provide evidence of the association between exposure to urban green and blue spaces (UGBS) and a range of health and well-being outcomes, as well as reducing health inequalities.^1^,^5^ Fairly limited knowledge on mechanistic pathways of UGBS outcomes suggests that time spent in UGBS provides restoration and relaxation and promotes social connection, which can improve mental well-being, self-efficacy and reduce mental ill-health.^6^,^9^ The spaces can also promote physical activity (including active travel),^1 4^ which is a known risk factor for non-communicable disease (NCDs). UGBS exposure has also been associated with benefits for maternal and child health, including improved birth weight, reduced incidence of gestational diabetes and eclampsia.^10^,^14^ Recent evidence also shows the benefits of UGBS exposure for reducing the risk of infections, including during the COVID-19 pandemic.^6 15^

However, the evidence base for the benefits of UGBS is largely founded on cross-sectional evidence, showing correlational associations between health outcomes and UGBS, with few longitudinal or interventional studies showing the causal effects of people using UGBS.^4 7 16^ Furthermore, the systematic review by Hunter *et al*^4^ showed that when evaluation of UGBS intervention studies was undertaken, rarely was a long-term (ie, beyond 1 year) follow-up conducted. More recently, a similar lack of longitudinal and intervention studies was found for UGBS and child and maternal health.^14^ Further, the lack of longitudinal and intervention studies means they have been of limited value in establishing causality. This study will contribute to this evidence gap with longitudinal data.

There are also important social patterns to UGBS exposure and associated health outcomes. Evidence suggests that the health benefits from UGBS exposure in low-income communities are attenuated due to less provision, accessibility challenges and poorer quality amenities, compared with the experience in high-income communities.^17^,^19^ Low-income communities are also disproportionately burdened with higher rates of NCDs and other health conditions and may be less willing or able to access high-quality UGBS.^17 18^ The issue of inequalities has been relatively underexplored in economic evaluations.^16^ This underscores the importance of high-quality longitudinal studies that leverage a wide variety of data (both primary and secondary) to reflect the multidimensional nature of the interconnections between health outcomes and socioeconomic status.

Previous research has also shown co-benefits of UGBS interventions for social, economic and environmental outcomes.^4 7 16^ Examples of social benefits include reduction in crime, increased perceptions of safety and enhanced sense of community belonging, while environmental benefits include increased biodiversity, reduced illegal dumping and climate adaptation such as urban cooling. Studies further demonstrated economic benefits, based on social return on investment (SROI) analyses,^20 21^ and increased footfall for businesses and higher house prices.^22^

In summary, despite considerable promise for improving health and well-being through the provision and use of UGBS, there is a need to extend our knowledge of the long-term public health outcomes of UGBS interventions, and of the co-benefits for environmental, social, economic and inequality outcomes. The Connswater Community Greenway (CCG) provides an opportunity for this, via a natural experiment evaluation of long-term outcomes. A research team led by the Centre for Public Health, Queen’s University Belfast has been conducting research on the CCG since 2010. This research primarily involved a before and after household survey using a repeated cross-sectional design in 2010/2011 (pre-implementation of the CCG) and 2016/2017 (6 months post implementation of the CCG) with 1200 local residents living in electoral wards within 1 mile of the CCG.^23 24^ The evaluation at 6 months post implementation showed small improvements in quality of life and mental well-being and suggested that the CCG may have mitigated population level decline in physical activity levels.^24^

A further SROI analysis^21^ demonstrated that the CCG was a viable investment, evidencing positive externalities across the domains of property values, flood alleviation, tourism and air pollution. Through the UK Prevention Research Partnership funded GroundsWell consortium,^25^ we now have an opportunity to conduct a long-term follow-up, 5 years post implementation. This long-term evaluation will facilitate investigation of the impacts on NCDs, such as incidence of mental health conditions, chronic respiratory conditions, type II diabetes mellitus and cardiovascular disease. It will also examine NCD risk factors including physical activity, and the impact on health inequalities. Moreover, it will allow us to deploy techniques and use data that previously have not been used to examine the costs and benefits of UGBS, as well as the distribution of these across sociodemographic groups. This paper describes the protocol for this 5-year follow-up of the UGBS intervention, the CCG, on a range of public health outcomes, as well as environmental, social and economic outcomes.

### Aims and objectives

The aim of this study is to evaluate 5-year public health and associated outcomes of the impact of a UGBS intervention, the CCG.

Our objectives are grounded in the following foundations and underlying assumptions:

- Understanding systems: the CCG and its impacts are bounded by, and dynamically interact with, the systems of which it is a part of or links to.
- Investigating outcomes, including economic impacts and processes.
- Synthesising and triangulating the evidence from a range of data sources and findings from the multiple analysis strands.

We broadly frame our objectives and methods using the following themes: systems, outcomes, processes, context, economic impact and evidence synthesis.

Objectives include to:

1. Codevelop an understanding of the systems context of the CCG using a range of systems science methods including soft systems methodology, causal loop diagram (CLD) and viable systems modelling.
2. Investigate the impact of the CCG on NCDs, co-benefits and inequalities using household surveys and longitudinal administrative data analyses.
3. Understand the influence of contextual factors, explain findings and clarify putative causal mechanisms of the CCG using qualitative and observational methods.
4. Investigate the economic impact of the CCG using SROI and cost-effectiveness methods.
5. Synthesise the evidence from the above evaluation through refinement of the draft CLD and participatory weight of evidence approach.

Details of the different methods and related statistical analysis for each aspect of the research are described below.

## Methods and analysis

### Study design

**Figure 1:**
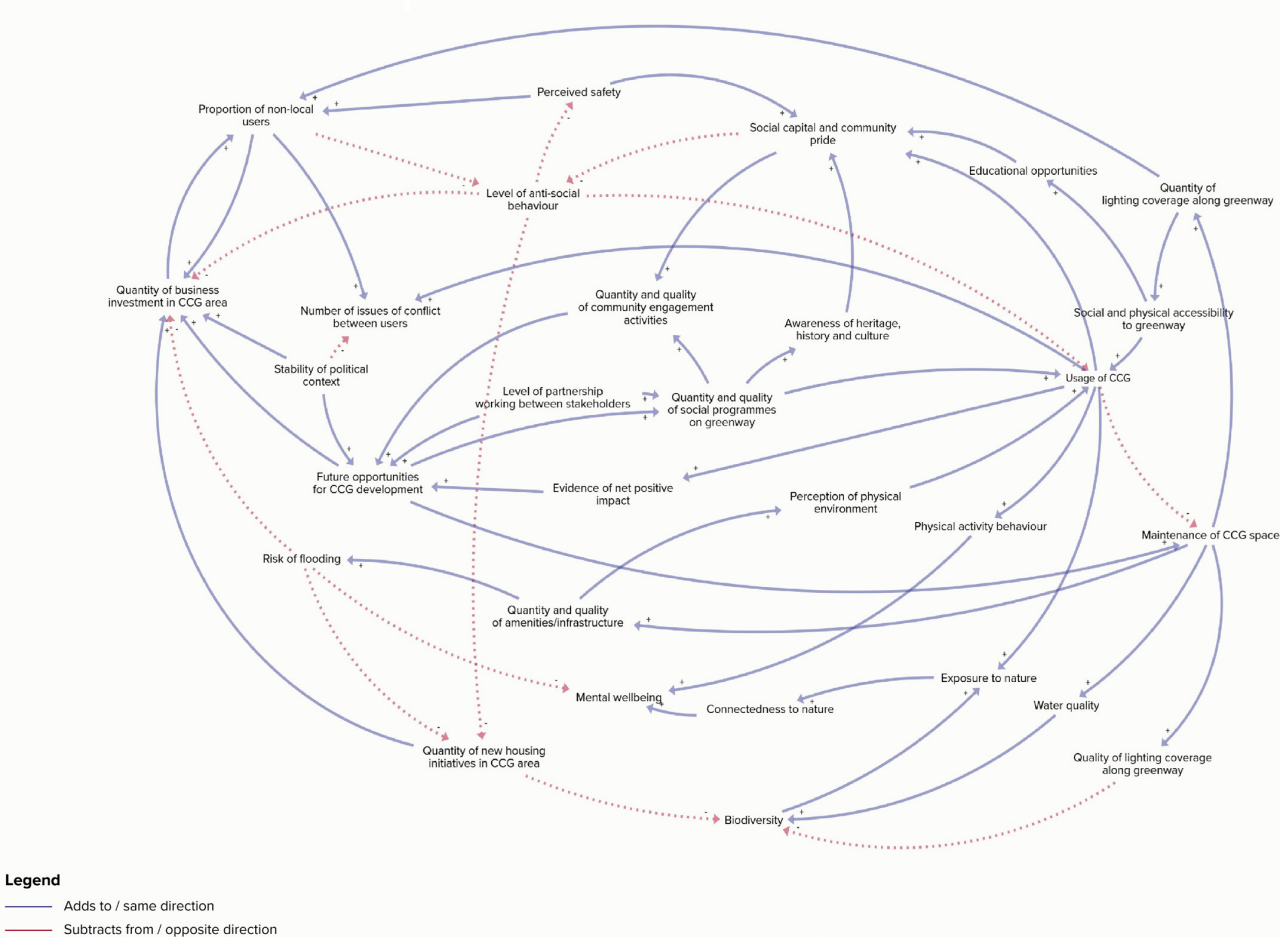
Causal loop diagram depicting the impacts of the CCG. Blue line=adds to/same direction; red dashed line=subtracts from/opposite direction. CCG, Connswater Community Greenway.

This natural experiment evaluation uses a range of system-oriented and mixed-methods techniques. A 1-day workshop in August 2022 was held with 23 stakeholders that included local community groups, local authorities, government departments, urban designers, landscape architects and local residents. The purpose was to discuss the public health impacts of the CCG 5 years post implementation. The workshop used group-model building techniques (Hovmand, 2014) to codevelop a CLD that depicted the plausible causal pathways linking the CCG and its impacts (see figure 1). The CLD was used to develop our 5-year follow-up study. For example, measures of biodiversity, sense of community pride and connectedness with nature were included in our evaluation as these factors were identified as important outcomes during the group-model building workshop. Data collection began in March 2023 and will be completed in September 2025.

### Intervention

The implementation of the CCG (https://www.eastsidegreenways.com/) provides the opportunity for a natural experiment evaluation of the public health impact of a major urban regeneration project (a new urban greenway) in Belfast, UK.

The aim of the regeneration project was to offer enhanced opportunities for physical activity and outdoor recreation through specific environmental improvements including the construction of 19.4 km of new cycle and walkways, and the provision of allotments. In addition, the regeneration aimed to improve the aesthetics of shared public spaces, involving the planting of trees and shrubs, erection of public art and the remediation of water courses to improve the natural diversity and reduce the risk of flooding. Along with the range of environmental improvements, several interventions to promote physical activity in the CCG area were implemented. Examples included the extension of neighbourhood walking groups and other physical activity initiatives to promote use of the Greenway, schools-based initiatives and community-based social marketing programmes. The development of the CCG began in 2011 and it was fully opened to the public in 2017.

### Intervention area

There are 29 district electoral wards (ie, spatial units used to elect local government councillors) in the political constituency of the CCG with a total population of approximately 110 600, and 22 wards (approximately 87 500 residents) with a geographical centroid within a 1-mile radius. Seven of these wards are within the top 25% most deprived wards in Northern Ireland, as determined by the Northern Ireland Multiple Deprivation Measure (NIMDM).^26^

### Population

As with our previous research on the CCG,^23 24^ our evaluation primarily focuses on adults (aged ≥16 years) living in the electoral wards whose geographical centroid is within a 1-mile radius of the CCG. For comparison purposes, the study also included people from other areas within the wider Belfast Metropolitan Area with a population of over 600 000.

### Understanding systems

A series of system-based modelling techniques is used to understand the complex system governing the development, implementation, management and maintenance of the CCG. UGBS interventions are increasingly perceived as complex systems requiring investigation and planning through a system lens. We will develop a systems-based model to represent the complex system governing the CCG from differing stakeholder perspectives. The proposed approach employs an adapted soft systems methodology^27^,^29^ process to explore the perspectives and needs of stakeholders and to inform the regeneration, sustainable development and maintenance of UGBS through a range of sequential integrated methods. Initially, we will generate a shared understanding of the context and use of the CCG through formal collaborative conversations, surveys and workshops with approximately 30 stakeholders from local community groups, local authorities, government departments, urban designers, landscape architects and local residents. Methods will include rich pictures,^27^,^30^ multiperspective diagrams,^31^ context diagrams,^30^ root definitions,^27^,^29^ stakeholder network analysis survey^32 33^ and the development of CLDs based on group model building methods.^34 35^ We will then model the contribution of stakeholders to the development, implementation and maintenance of the CCG with the viable systems model,^36^,^39^ informed by soft systems methodology conceptual models and the CLDs. Critical dependencies will be determined by generating and analysing a dependency model.^40^ Measures of success can be elicited from the soft systems methodology conceptual model activity analysis and analysis of the CLDs (see table 1 for descriptions of systems science methods). These proposed approaches can help decision-makers and planners identify the stakeholders required for each phase, their roles and responsibilities and build capacity in local communities to develop amenities to meet their needs. The CCG will be modelled in two phases: (1) development and implementation; and, (2) management and maintenance. A ‘Reference Model’ is then derived from integrating the data generated by the multiple systems methods used (soft systems methodology conceptual model, viable systems model, CLD and dependency model). The outputs will be developed iteratively, leading to a multiperspective system model integrating the various methods.

**Table 1:** Definitions of systems science methods

Method	Description	Reference
Causal loop diagrams	Causal loop diagrams can be used to show the relationships between causal factors and how they operate within a system (or systems).	Stroh,^34^ Vandenbroeck *et al*^35^
Context diagrams	Context diagrams are graphical representations of the system that include a series of concentric circles to identify the boundaries of the system and the level of our influence on the system. The context diagramming approach can help stakeholders understand the system being studied, depict its boundaries and facilitate the development of shared mental models.Drawing a context diagram, or a context analysis, is a way of organising thinking about factors that characterise a ‘problematic situation’ and is prepared from the perspective of a particular actor, mostly stakeholders, about a problematic situation.	Flood and Carson^30^
Dependency model	Dependency modelling is a practical approach to articulate and understand the project’s overall purpose, connect project objectives and goals and form a language to discuss risks among stakeholders and the project. This approach is based on goals, objectives and the prerequisites to satisfy interconnected goals.	Slater^40^
Multiperspective diagrams	Diagrams represent the range of perspectives within the communities and other citizen groups, such as the elderly, young families and youth.	Dodd and Alston^31^
Rich pictures	A visual way of building a picture of the collective views and perspectives of those involved. It can be used to define the problem and identify the systems impacting the problem and any solutions. A rich picture is a suitable method for capturing information and data collaboratively from groups of people and stakeholders and extracting their beliefs or understanding of an issue, situation or problem.	Checkland and Poulter,^27^ Checkland and Scholes,^28^ Flood and Carson,^30^ Wilson^29^
Root definitions	A root definition is a detailed paragraph that describes the system to address the problematic situation, project or system of interest. Root definitions are based on shared understanding from a range of stakeholders (such as users, project leaders) to describe the purpose and outcomes of the system of interest or problematic situation.	Checkland and Poulter,^27^ Checkland and Scholes;^28^ Wilson^29^
Soft systems methodology	Soft systems methodology (SSM) was initially introduced by Checkland (1981) as an organised way of thinking in problematic social situations and change management. This traditional SSM approach, called Checkland-based Soft Systems Methodology, requires continuous revisions to produce change through dialogue and is not perceived as a practical way of fulfilling all organisational needs. Brian Wilson further enriched and developed this method. Under Wilson’s approach, a comprehensive consensus on organisational purpose is achieved through a detail-oriented approach, and more extensive system models are developed. Subsequently, a wider range of data, such as measures of performance, environmental constraints and organisational roles and responsibilities, is analysed to reach a consensus on a blueprint of an organisation that can fulfil organisational needs and processes.	Checkland and Poulter,^27^ Checkland and Scholes,^28^ Wilson^29^
Stakeholder network analysis	Stakeholder network analysis is the exploration of the interactions between individuals, organisations or stakeholders, and the relationships between them. It provides a set of theories, techniques and tools for understanding structural and relational aspects as stakeholders and organisations interact with others.	Nigg *et al*,^32^ Provan *et al*^33^
Viable systems model	Viable system model can be employed as a framework for organisation design to identify where the activity functionally sits in the project, diagnose organisational weaknesses, mismatches or missing elements and resolve the analysed issue organisations.	Beer,^36^ Beer,^37^ Espejo and Reyes,^38^ Leonard^39^

### Investigating outcomes

#### Household survey

We will conduct a random household survey of∼1200 adults living within the sampling area of the CCG to collect information on health, mental well-being, social capital and perceptions of the environment. There have been two previous waves of the household survey: wave 1 in 2010/2011 (n=1037) before the development of the CCG began; and wave 2 in 2016/2017 (n=968) 6 months after the completion of the CCG. A random sample of households in the Connswater area was invited to participate in a survey at both time points to assess attitudes towards, and levels of, habitual physical activity, perceptions of the characteristics of the environment associated with active travel and physical activity and individual and social networks and their potential influence on behaviour.^23^ A repeat survey (wave 3) was conducted in 2023/2024 to provide a 5-year long-term evaluation of the regeneration project. We used a repeated cross-sectional design as we investigated population level impacts.

The household survey is intended to determine the impact of this system-wide community intervention on physical activity in the context of a major inner city urban regeneration project. We have designed this study to be comparable across the three waves and to other Northern Ireland surveys of physical activity, providing a context for any changes observed in the population. Having a validated measure of physical activity at each wave will enable us to determine the effects of the environmental changes on physical activity levels.

As with the first two waves, participation in wave 3 will be voluntary, with minimal exclusion criteria applied. Households will be randomly selected to participate using addresses drawn from the Land and Property Services list and will be sent a letter describing the study (see below).

This element of the study will evaluate the long-term public health and co-benefit effects of an urban greenway in a deprived area of Belfast, Northern Ireland using a quasi-experimental before and after survey of the intervention population (defined below). The data will be compared with those from a parallel (before and after the implementation of the CCG) survey of comparator areas across Northern Ireland, including Belfast and areas with similar population density and deprivation characteristics as the CCG area, conducted by the Northern Ireland Statistics and Research Agency.

Our primary outcome for this third household survey is physical activity as defined during the community consultation for wave 1. A sample size calculation was performed based on the estimated proportion of the population achieving the minimum recommended level of physical activity (at least 150 min of moderate-to-vigorous physical activity per week), using alternative assumptions of 20%, 30% and 50% achieving recommended levels. Estimates of the sample sizes required to detect differences in population proportions, assuming effect sizes of 0.15, 0.20 and 0.25 are 934, 526 and 337, respectively. We will therefore aim to invite 1200 adults (aged ≥16 years of age) for wave 3, assuming a 20–30% response rate.^23^

A list of 1200 households in the CCG area will be randomly sampled from the Land and Property Services list. The ‘next birthday rule’ will be used to identify who in the household will be invited to complete the survey. Each of the 1200 households will receive an invitation to participate along with a Freephone telephone number for them to contact Perceptive Insight Market Research Limited to make an appointment for an adult (≥16 years of age) to be interviewed. Interviewers from Perceptive Insight Market Research will visit in-person to each household (including those who have not made contact) to recruit an adult (≥16 years of age) from each household to participate. Up to four in-person visits to the household will be made to achieve an interview. When visiting the participant in their home, following a brief explanation of the study and providing participants with the participant information sheet, participants will be asked to provide written informed consent before commencing the survey. The same recruitment practices were used in previous waves. For those who receive the invitation and do not want to participate, they can contact the company to let them know. Data will be collected over a 12-month period to account for seasonality.

The analyses of the three waves of survey data will assess: (1) attitudes towards and levels of habitual physical activity, using the Global Physical Activity Questionnaire; (2) change in neighbourhood walkability using a validated survey instrument that measures perceptions of aesthetics, green space, access to amenities, convenience, traffic and safety; (3) changes in social capital as reflected in civic engagement, neighbourliness, social networks and support, perceptions of local area and sense of community pride; (4) change in Common Core Theoretical Constructs such as intention; (5) change in mental well-being measured using the Warwick Edinburgh questionnaire; (6) change in general health measured using the Short Form 8 (SF-8) questionnaire; (7) change in quality of life measured using the EuroQol-5D (EQ5D) questionnaire; (8) demographic and lifestyle characteristics; (9) use of the CCG; and (10) engagement with the natural environment (see online supplemental file 1).

#### Administrative data

We aim to undertake longitudinal administrative data analyses to investigate the impact of the CCG on NCDs and inequalities. Administrative data includes routinely collected records from 2010 to 2023 by National Health Service (NHS) on death, hospital admissions, prescribed medications, pregnancy and birth. Further details of the administrative data sources and variables are provided in online supplemental file 3. Given the 14-year follow-up time frame (5-year post implementation), the administrative data will enable us to investigate the impact of the CCG on outcomes like NCD incidence which requires time to occur. We will also investigate the impact of the CCG on infections, and child and maternal health outcomes. The analyses will focus on NCDs including mental ill-health (eg, depression, anxiety), cardiovascular diseases (eg, hypertension, stroke), type II diabetes mellitus, chronic respiratory diseases (eg, chronic obstructive respiratory disease, asthma) and death and infection (bacterial and viral) rates.

During wave 3 data collection of the household survey, participants will be asked to provide written informed consent to link their survey data to administrative data using personal information (ie, name, date of birth and address) via the Honest Broker Service, Northern Ireland. This will facilitate household data linkage at the individual level, enabling detailed investigation of NCD outcomes (eg, depression, anxiety) from the administrative data and NCD risk factors (eg, physical activity, social environment) from the household survey. Online supplemental file 2 outlines the process for linking household survey data and administrative data.

#### Outcomes

The primary outcome will be the change in the proportion of the population meeting the current recommended target of 150 min of moderate intensity physical activity per week between wave 1 (baseline) and wave 3 (5-year follow-up), measured using the Global Physical Activity Questionnaire.

Secondary outcomes include:

- Change in mental well-being measured using the Warwick Edinburgh Mental Well-being Scale.
- Change in general health measured using the SF-8.
- Change in quality of life measured using the EQ-5D.
- Changes in social capital as reflected in civic engagement, neighbourliness, social networks and support and perceptions of local area.
- Change in Common Core Theoretical Constructs such as intention.
- Change in neighbourhood walkability using a validated survey instrument that measures perceptions of aesthetics, access to amenities, traffic and safety.
- Use of the CCG.
- Engagement with the natural environment.
- Demographic and lifestyle characteristics.
- Mental ill-health (measured by medication prescriptions (eg, antidepressants, anxiolytics), admissions to the emergency department, deaths).
- Cardiovascular disease (measured by medication prescriptions (eg, aspirin, ACEs, beta blockers, glyceryl trinitrate, statins), admissions to emergency department, deaths).
- Type II diabetes mellitus (measured by medication prescriptions, admissions to emergency department, deaths).
- Chronic respiratory diseases such as asthma (measured using medication prescriptions, admissions to emergency department, deaths).
- Bacterial and viral infections (measured by medication prescriptions (eg, antibiotics)), including COVID-19 infection rates.
- Birth outcomes (measured by prenatal and neonatal health outcomes for both the mother and the new-born including gestational age, birth weight, Apgar score and postpartum depression).
- Deaths (measured by all-cause mortality).
- Health inequalities—subgroup analysis by NIMDM quintiles.

#### Statistical analysis

The intervention group is defined as those living in the 29 electoral wards with a geographical centroid within a 1-mile radius of the CCG. We define three comparator groups:

1. Other super output areas within the Belfast Metropolitan Area.
2. Distance decay analysis with those living <400 m, <800 m, <1200 m from the CCG based on distance from household (using the Unique Property Reference Number) to nearest accessible point of the CCG using a digitised footpath network developed in our previous research^20 21^ for the administrative data. We will use the x,y co-ordinates for the individual household addresses for the household survey.
3. Matched super output areas across Northern Ireland based on deprivation, urban–rural classification and population density.

#### Study time frames

- Pre-2011: baseline/pre-implementation of the CCG.
- 2017: immediately post-implementation of the CCG.
- 2022/2023: 5 years post implementation of the CCG.

#### Analytical approach

For the administrative data, we will evaluate difference-in-differences in outcomes (intervention area vs comparator areas) across three different time series (baseline (2010/2011), year 1 post implementation (2016/2017) and year 5 post implementation (2022/2023)).

Our difference-in-differences models will include an interaction term between study area (eg, intervention, matched control) and time period (preintervention and postintervention), which will allow us to formally test for differences in trends. This method implicitly adjusts for any secular trends that may influence the risk of NCDs under the assumption that these act identically across the intervention and comparator areas.

For the household survey data, regression-based modelling approaches will be used to calculate the mean difference at baseline (in 2010) compared with postintervention (in 2017 and 2023) (and 95% CI) after adjusting for confounders. Multilevel models will be fitted using a random intercept at the super output area (individuals within super output areas) to account for clustering within areas. For the administrative data, the difference in odds (medication)/hazard (diagnosis) between the baseline, intervention and follow-up periods will be calculated.

The analyses will be stratified by distance from the CCG of those in the intervention group (exposure to the intervention) and by deprivation. Interaction tests will be conducted by fitting interaction terms within regression models. We will explore the impact of length of time resident in the intervention area in a sensitivity analysis.

To investigate effects on health inequalities, we will undertake a stratified analysis to assess whether any impacts on the outcomes are socially patterned by socioeconomic status, age and sex informed by the PROGRESS-Plus framework (Place of residence, Race, Occupation, Gender, Religion, Education, Socio-economic status, Social capital).^41^ We will assess whether the differences in the social patterning of outcomes changed over time post-intervention compared with baseline.

We will also conduct mediation analyses to explore plausible mechanistic pathways between UGBS, NCD risk factors and NCDs using structured equation modelling approaches based on previous published analyses.^7 8^

### Process

#### Qualitative exploration

We will conduct a series of community focus group workshops to explore the health, social, economic and environmental impacts and mechanistic pathways of the CCG using rich pictures, multiperspective diagrams, CLDs and focus group discussions. We will conduct two sequential workshops with adults (aged ≥16 years) living within a 1-mile radius of the CCG. Workshop one will involve focus group discussions and system-thinking methods (eg, rich pictures, multiperspective diagrams) to stimulate conversations within the group, assist with sense-making and enable participants to reflect on their own experiences, views and perceptions. Workshop two will involve validation of the analyses and findings with participants from workshop one. The research team will present the key findings from workshop one and an overarching rich picture.

Based on focus groups undertaken in the previous waves of the CCG evaluation,^42^ we estimate that we will need to recruit approximately 75 adult participants to reach data saturation in wave 3. Participants will be invited from those who have completed the household survey and through local community networks with partner organisations and purposively sampled by sex, age and super output area to recruit a diverse sample.

We will use focus groups to enable the dynamic exchange of thoughts and experiences between participants, enabling more insightful discussions. By following a structured line of questioning in the focus group discussions, we will codevelop multiperspective diagrams and rich pictures. In combination with the group discussions, participants will be asked to provide their thoughts, experiences and perceptions relating to the impact of the CCG by sketching them on paper. The development of rich pictures will help to communicate and articulate complex concepts and ideas. Rich pictures help in the exploration of thoughts by making sense of and mapping a system. Participants will be invited back to a second workshop where the research team will present an overarching rich picture from workshop one and undertaken during qualitative data analysis. Participants will be asked to provide feedback to the research team in relation to the accuracy of and inferences from the data analysis.

Data analysis will follow a thematic analysis approach, providing flexibility for the generation of themes as they emerge from the data. The first stage of analysis will be familiarisation with the data and coding. The codes and themes will then be discussed by the research team. Following the establishment of the coding protocol, analysis will continue to further develop the theme review (ie, naming and defining themes) and coding framework. In addition to thematic analysis, the research team will also review each of the rich pictures and the corresponding transcription. This will provide a more detailed understanding of how each group understands and has mapped the system. They will then work to analyse each of the rich pictures and to identify corresponding patterns and relationships within each of the rich pictures and work to determine how the dynamic complexities of the system have been understood by participants.

### Intercept surveys and direct observations

We will conduct a process evaluation of the use of the CCG using intercept surveys and observational methods. Direct observation of usage of the CCG will be conducted in two ways:

1. Conducting a third wave of intercept surveys of CCG use (at four locations on the CCG), adapting Sustrans methodology similar to previous waves which were conducted at the same time as the household surveys. The surveys will take place on four separate days at each of the four locations—16 survey days in total. The days for each site (07:00 to 19:00) will be: term time weekday; term time weekend day; non-term time weekday; non-term time weekend day. Surveyors will count all cyclists, e-bikes, pedestrians, horse riders, roller skaters and wheelchair users passing the survey point, covering people travelling in all required directions—including those travelling on the neighbouring pavement and on-road cycles. Other data collected will include route choice, distance travelled, perceptions of safety, physical activity and mental well-being.
2. Employing the System for Observing Play and Recreation in Communities (SOPARC) methodology, a validated technique to assess the use being made of green spaces and parks in the area before and after the construction of the CCG.^43^ We will recruit and train volunteers from the local community to help with data collection at eight target areas using the SOPARC protocol, over the course of 7 days in winter and in summer. Analysis will explore the characteristics of CCG users, the types of activities they are involved in, their reasons for using the CCG and where they are travelling to/from to identify local users and visitors from outside the local community.

### Economic analyses

#### Cost-effectiveness analysis using administrative data

The economic evaluation will adopt a difference-in-difference approach to the quantification of costs and benefits arising from exposure to the CCG. Resource use will be based on cumulative drug costs and length of hospital stay (monetised using estimates of a bed day cost).^44^ The difference-in-difference in resource use will be estimated using a similar regression approach to that detailed above, in which distance to the CCG is interacted with time to estimate the effect on healthcare resource consumption. A generalised linear regression model will be employed to allow maximum flexibility over the functional form used. Any savings generated in terms of healthcare will be set against the cost of the CCG expressed in terms of their equivalent annual cost for the time period examined. The resultant net cost will constitute the value to be compared against benefits in terms of outcomes from an NHS perspective.

#### Cost-effectiveness analysis using household survey data

Conducting an economic evaluation of UGBS interventions that promote physical activity is fraught with methodological difficulties, and multiple approaches have been recommended,^16^ from which we will draw on several. First, we will use a cost-effectiveness approach and adapt the PREVENT model,^45^ collecting information about the costs of the CCG construction and interventions in the CCG area and the outputs in terms of the reduction in the proportion of the population categorised as physically inactive, thereby permitting us to derive incremental cost-effectiveness ratios (ICERs). The impact on ICERs of varying the model assumptions will be explored in subsequent sensitivity analyses. We will use similar methodology to that in our earlier modelling study.^46^

#### Cost-benefit analysis

Benefits will be estimated based on the difference in the difference in disease risk, where treated disease based on prescribed medicines and hospital inpatient stays are used to define presence or otherwise of a condition and differences in these the change in risk. Logistic regression models will provide the analytic approach where time and proximity to the CCG are the key regressors. We will adopt the approach set out by Robinson and Hammitt^47^ for valuing non-fatal health risk reductions based around willingness to pay estimates to value risk reduction. Reductions in mortality as estimated above will be used in conjunction with estimates of the statistical value of a life to estimate the value of reductions in fatal risks. These benefits will be set against the costs of the CCG for the period of observation and discounted to estimate the net present value of the CCG. There are a number of assumptions underpinning this approach, namely, we assume that the supply of healthcare is perfectly elastic with respect to need and/or that there exist only random effects with respect to where need originates geographically.

#### Social return on investment analysis

We will conduct an updated SROI analysis of the CCG framework previously presented by Hunter *et al*^20^ and Tate *et al*^21^ over an assumed lifetime of 40 years using data available pre-implementation (2012) and 5 years post implementation (2023). Using real-world data from three time points (2012, 2017 and 2023), our analysis will focus on key elements identified by stakeholders as part of the group model building workshop described earlier. These include: property values; flood alleviation; tourism; biodiversity; climate change; health and well-being; crime; and, employment and productivity. We will also explore the economic cost of illness and premature deaths attributable to air pollution and how this was reduced by the CCG.^21^ Using SROI analysis, we will estimate the value of the CCG over a 40-year horizon as this aligns with the commitment by the local authority to manage and maintain the CCG. Our analysis will use both primary and secondary data collected before and after the CCG was constructed. In line with recommendations from the HM Treasury Green Book,^48^ and the framework put forward by Deidda *et al*,^49^ we will report the estimated economic benefits of the societal dimensions of the CCG and compare these to the total costs of the project. We will describe various sensitivity analyses to explore the robustness of the results. Finally, we will explore the distributional effects of the CCG and how the various domains of the SROI model affect some subgroups, such as deprivation, by undertaking subgroup analyses.^50^

#### Decision theoretic approach

It is possible that the costs and elements of benefit are determined jointly. For example, as the level of investment in green space increases, so too does its quality and with it the level of benefit. In this situation, errors from cost and outcomes function may not be independent, and standard parametric approaches to estimation of cost-effectiveness may not be appropriate. We shall explore this, and if appropriate, adopt non-parametric methods based on a decision-analytic approach to inform recommendations about whether or not the intervention is likely to be effective.^51^ The decision-analytic approach will use relevant knowledge, theory and data from the empirical data to form a view on: (1) whether or not the intervention is likely to cause harm; (2) if not, whether or not, in the light of the (low) cost of an intervention and if it is likely to be effective enough to be cost-effective. The approach will address questions such as: (1) What level of effectiveness is required for UGBS interventions to be cost-effective or cost-beneficial, given the costs of the intervention? (2) Do the benefits identified in the CLD, but not measured in terms that can be incorporated in the cost-benefit or cost-utility analysis, such as changes in sense of community pride, reinforce or weaken these conclusions?

### Evidence integration and synthesis

We aim to synthesise the evidence from the above evaluation through refinement of the preliminary CLD and a participatory weight of evidence approach. The original CLD (figure 1) will be updated and refined by combining: (1) perspectives of multiple stakeholders collected using soft systems methods, viable systems modelling and participatory group model building methods; and, (2) findings from the above analyses. This combined approach should allow us to go beyond existing frameworks by integrating scientific and practice-informed evidence, systematically analysing underlying values, beliefs and mechanisms and explicitly considering feedback loops. A final group model building workshop will be conducted with 15–20 stakeholders (local communities, industry, practitioners, researchers, national-level and local-level government agencies) using the community-based system dynamics approach^52^ which will involve discussions to resolve divergences or convergences in the findings. An overarching conceptual framework will be created combining the stakeholders’ perspectives and findings from the above analyses, adapting a contribution analysis approach.^53^ We will challenge edges, chains of edges or subgraphs of the CLD initially using the evidence from the analyses detailed above.

To represent the generated evidence in an accessible way to stakeholders, we will use a participatory weight of evidence approach^54^ involving fuzzy cognitive mapping. Briefly, we will represent the evidence syntheses as cognitive maps, where contributing factors from quantitative data are linked to the outcome effect estimates from the above outcome analyses. Themes from the qualitative data will also be included.

#### Spatial microsimulation modelling

The aim of this aspect is to estimate NCD, economic, societal and health inequality impacts of the CCG, and to inform possible interventions and programmes to ensure future impact. We will codevelop a data-informed spatial microsimulation model in which the data from the above evaluation will be integrated for subsequent in silico experimentation to inform future policy action. In line with ecological models,^55^ the model will account for the social environment, urban landscape design and inequalities in access to and quality of UGBS, as well as individual residents’ attributes (including demographics and health-related behaviour) that may affect the impact of the CCG. The data-informed microsimulation model will draw strongly from the findings described in the analyses detailed above and secondary data analysis.

### Patient and public involvement

The study has engaged extensively with local residents since research began on the CCG in 2010. This includes quarterly research updates and discussions with a stakeholder’s forum led by EastSide Partnership (who are responsible for developing and managing the CCG). Membership (over 100 members) includes local residents and multisectoral stakeholders involved in the design, development, implementation and management of the CCG. Consultation and engagement with the local community have helped to shape the development of the research programme. For example, group model building workshops involving the local community led to the inclusion of biodiversity, nature connectedness and sense of community pride measures being included in the wave 3 household survey and further explored in the qualitative workshops.

## Discussion

Previous research has highlighted the gap in knowledge regarding long-term impacts of UGBS interventions.^4 7 16^ The current study aims to address this knowledge gap. This paper presents a protocol for an evaluation of a 5-year follow-up investigation of the public health impact and co-benefits of an urban greenway. The study will use a variety of systems approaches along with other qualitative and quantitative methods. The evaluation will focus on outcomes such as the incidence of NCDs and NCD risk factors, and other environmental, social and economic outcomes, as well as the impact on health inequalities.

We will extend our previous research^23 24^ by conducting this 5-year follow-up evaluation of the impact of the CCG, incorporating new dimensions including outcomes on NCDs, sense of pride and biodiversity, informed by the CLD, incorporation of administrative data and qualitative research methods. Integrating a range of methods and comparator strategies in our analyses reduces the risk of bias in this study.^56 57^ The research has potential to inform future research, policy and practice on UGBS interventions for public health impacts and co-benefits.

## Supplementary material

10.1136/bmjopen-2024-097530 online supplemental file 1
