# Supplementary material for: Investigating the long-term public health and co-benefit impacts of an urban greenway intervention in the UK: a natural experiment evaluation – study protocol
Source: BMJ Open. 2025 Jul 6;15(7):e097530. doi: 10.1136/bmjopen-2024-097530 (PMC12230955; doi:10.1136/bmjopen-2024-097530)
Supplement: online supplemental file 1 [file bmjopen-15-7-s001.docx]

**Supplementary File 1: Table of outcomes for the household survey**

| **Constructs** | **Measures** |
| --- | --- |
| Physical activity | Global Physical Activity Questionnaire |
| Health and wellbeing | Warwick Edinburgh Mental Wellbeing Scale  Short-Form 8 – general health  EuroQol 5D – quality of life |
| Individual factors | Age, sex, marital status, accommodation, weekly household income, education level, number of children in household, height/weight, general health, car in household, adult bicycle in household, employment status |
| Psychological factors | Intentions, readiness to change, physical activity self-efficacy, physical activity outcome expectancy |
| Community factors | Social capital (local area trust, social networks, community participation) |
| Physical environment | Perceptions of environment (aesthetics, traffic, safety, amenities) |

Co-developed in consultation with local multi-sectoral stakeholders and piloted with n=100 residents

**Supplementary File 2: Consent to data linkage process between the household survey and administrative data**


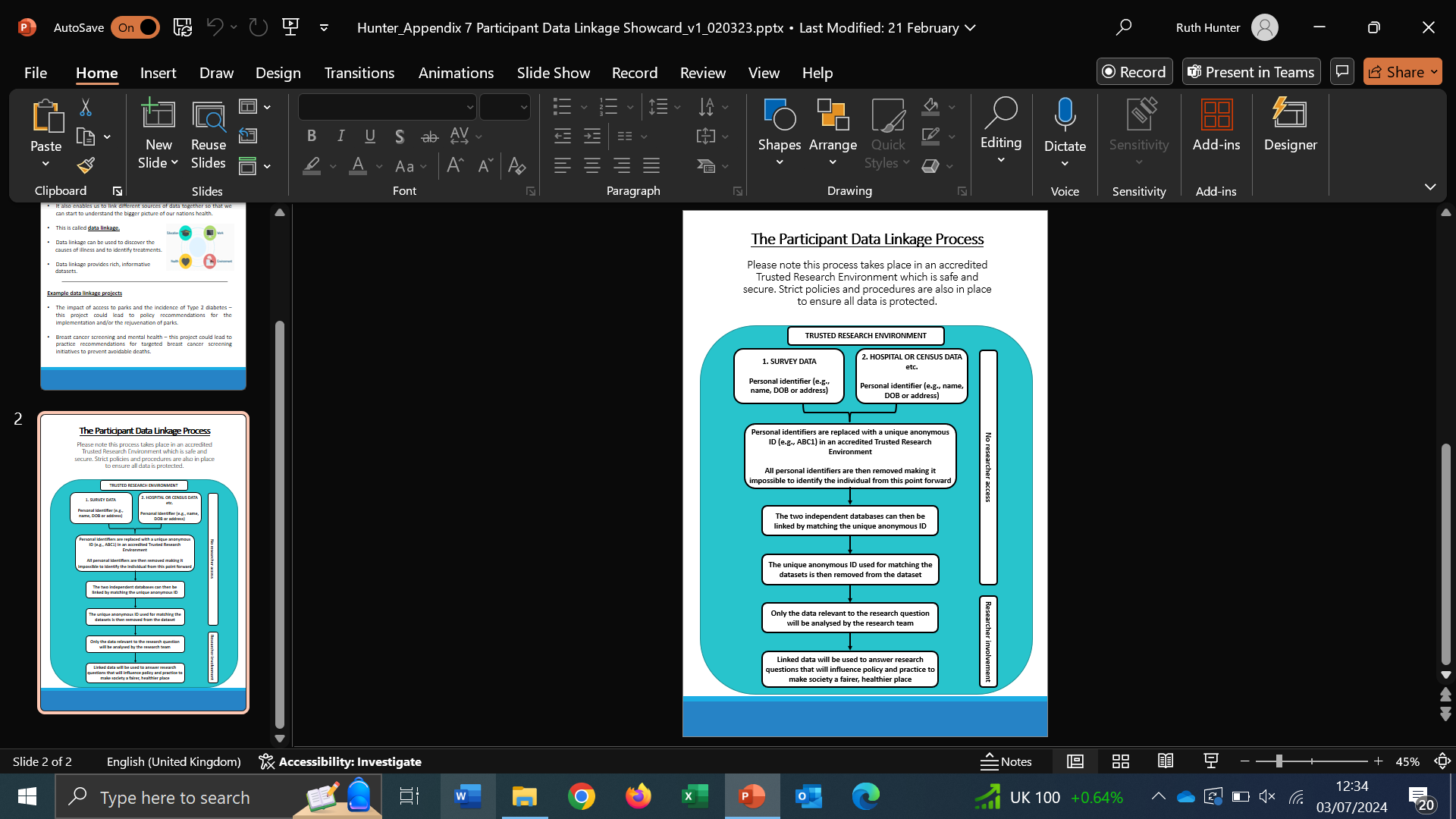


**Supplementary File 3: Details of Administrative Data Sources and Variables**

Diseases coded using the 10th Revision of International Statistical Classification of Diseases, version 2018. For the economic analysis the length of hospital stay will be used.

**Administrative Data**

**National Health Application and Infrastructure Services (NHAIS) data**
NHAIS contains information on demographics (e.g., gender and age), health and care number, property reference number (UPRN) (only assigned in BSO), and geographical identifier super output area (SOA) (only assigned in BSO). Health and care number will be used to link to other health data held by BSO, and UPRN will be used to link to Land and Property Services (LPS) data. The identifier of SOA will be used to link individuals to Northern Ireland Multiple Deprivation Measure (NIMDM) 2017. Finally, health and care number, UPRN, SOA will be removed from the researcher dataset.

**Enhanced Prescribing Data (EPD)**

Mental ill-health: Potential mental ill-health will be identified using receipt of psychotropic medication (Maguire et al., 2016). All psychotropic medications dispensed in community pharmacies between January 2010 & Most recent will be collected from EPD, which will be linked to NHAIS samples by health and care number.

The following BNF sections identify psychotropic medications: BNF 4.1.1 Hypnotics, BNF 4.1.2 Anxioltyics, BNF 4.1.3 Barbiturates, BNF 4.2.1 & 4.2.2 Antipsychotics, BNF 4.2.3 Mania and hypomania, BNF 4.3.1 Tricyclic Antidepressants, BNF 4.3.2 MAOI Antidepressants, BNF 4.3.3 SSRI Antidepressants, BNF 4.3.4 Other Antidepressants.

For ADHD, the following BNF sections identify relevant medications: BNF 4.4 Atomoxetine & Dexamfetamine Sulfate & Lisdexamfetamine Mesilate & Methylphenidate Hydrochloride

Dementia: For Dementia, the following BNF sections identify relevant medications: BNF 4.11 Drugs for Dementia.

Cardiovascular disease: For cardiovascular disease, the following BNF sections identify relevant medications: BNF 2.12 Lipid Regulating Drugs (Statins): only atorvastatin, fluvastatin, pravastatin sodium, rosuvastatin, simvastatin; BNF 2.4 Beta-adrenoceptor blocking drugs; BNF 2.5.5.1 Angiotensin-converting enzyme inhibitors (ACEs); BNF 2.5.5.2 Angiotensin-II receptor antagonists (ARBs); BNF 2.6.1 Nitrates (e.g. GTN).

Type II Diabetes Mellitus: For Type II diabetes mellitus, the following BNF sections identify relevant medications: BNF 6.1.2.2 Biguanides (Metformin).

Chronic respiratory disease: For chronic respiratory disease, the following BNF sections identify relevant medications: BNF 3.1.1 Adrenoceptor agonists (e.g. SABA/LABA & other adrenoceptor agonists); BNF 3.1.2 antimuscarinic bronchodilators (e.g. LAMA); BNF 3.2 corticosteroids (e.g ICS); BNF 3.3.2 leukotriene receptor antagonists (e.g. LTRA); BNF 3.4.2 omalizumab.

Infections: For bacterial infections, the following BNF sections identify relevant medications: BNF 5.1.1 Penicillins (or just BNF 5.1.1.3 Broad spectrum Penicillins); BNF 5.1.5 Macrolides (e.g. erythromycins).

For viral infections, COVID-19 related datasets include Pillar 1 (HSC) testing and Pillar 2 (National Programme) testing. Data will be linked to NHAIS population spine using HCN. COVID-19-related information includes whether and when the individuals tested positive (From hospital records and self-report)

Monthly DDD for each medication for each of the ~160 study months Jan 2010-Most recent will be a proxy of uptake and severity. Variables including ‘Scan_month’, ‘Scan_year’ will be used to identify the time of prescriptions.

Birth outcomes

Data on birth outcomes from 2011 to present will be obtained from the Northern Ireland Regional Maternity System (NIMATS). NIMATS collects data on all births recorded in Northern Ireland, including information on the mother, the baby, and the pregnancy. The following variables within NIMATS will be used as birth outcome indicator: Mothers: Date of birth, age group at birth, employment, height, weight, marital status, ethnic group, country of birth, pre-existing health conditions such as blood pressure, diabetes type, diabetes profile, respiratory disease, mental health, COVID-19 status, COVID-19 vaccination status, first language description, family support, smoking, alcohol use, sleeping patterns. Pregnancy: parity, number of previous pregnancies

Infants: date of birth (year and month), sex, Apgar score, birthweight, birth status, head circumference, gestation at delivery, hospital duration at birth (including NICU stay). Partner: employment, height, weight, smoking. Using continuous variables such as birth weight and gestational age in weeks, we will be able to obtain categorical birth outcomes including very low birth weight, low birth weight, normal birth weight, excessive birth weight, term birth, preterm birth, and very preterm birth

**Hospital Inpatient Data (PAS)**

Relevant hospital inpatient data between January 2010 & Most recent will be collected from PAS, which will be linked to NHAIS samples by health and care number. Data includes month of admission, year of admission, month discharge, year discharge, and relevant ICD-10 codes for reason for admission. ICD-10 codes will be grouped into a range of health conditions including: 1) Cardiovascular diseases (fatal and non-fatal) 2) Mental ill-health such as Depression (diagnosed by a physician, hospital discharge register or any validated scale with a cut-off for depressive symptoms), anxiety, ADHD 3) Dementia (consensus diagnosis made according to national or international guidelines) 4) Chronic respiratory disease including COPD and asthma 5) Type II Diabetes Mellitus 6) Bacterial and viral Infections Diseases coded using the 10th Revision of International Statistical Classification of Diseases, version 2018. For the economic analysis the length of hospital stay will be used.

**Emergency Department Data (Symphony)**

**General Register Office Northern Ireland' (GRONI) data**
GRONI death data will be used to determine fact of and cause of death. We require all primary cause of death, which will be provided as ICD codes, and we will categorise them into subgroups for analysis. Variables including ‘Month Death’, ‘Year Death’ will be used to identify the time of death. ‘Cause of death',

**External data**

**NINIS data**
Northern Ireland Multiple Deprivation Measure (NIMDM) 2017 will be treated as categorical variable (least deprived to most deprived) to identify area-level deprivation. This variable will be operated as quintiles. SOA identifier will be used to link NIMDM to NHAIS samples.

**Land and Property Services (LPS) data**
House value (capitalvaluenonexempt) from 2020 LPS data will be used to assign a house value category to NHAIS samples by UPRN. This variable will be operated as quintile (1^st^ quintile: <£99,999, 2^nd^ quintile: £100,000-£199,999, 3^rd^ quintile: £200,000-£299,999, 4^th^ quintile: £300,000-£449,000, 5^th^ quintile: >£450,000). This will be used in analysis as an indicator of socio-economic status.
