# Supplementary material for: Investigating the long-term public health and co-benefit impacts of an urban greenway intervention in the UK: a natural experiment evaluation – study protocol
Source: BMJ Open. 2025 Jul 6;15(7):e097530. doi: 10.1136/bmjopen-2024-097530 (PMC12230955; doi:10.1136/bmjopen-2024-097530)
Supplement: Supplementary file 2 [file bmjopen-2024-097530.reviewer_comments.pdf]

PEER REVIEW HISTORY

BMJ Open publishes all reviews undertaken for accepted manuscripts. Reviewers are asked to complete a checklist review form and are provided with free text boxes to elaborate on their assessment. These free text comments are reproduced below.

ARTICLE DETAILS

Title (Provisional)

Investigating the long-term public health and co-benefit impacts of an urban greenway intervention in the UK: A natural experiment evaluation – Study protocol

Authors

Hunter, Ruth F.; Cleland, Claire; Wang, Ruoyu; O'Neill, Ciaran; Mullineaux, Shay; Tate, Christopher; Küçükali, Hüseyin; Akaraci, Selin; O’Kane, Niamh; Garcia, Leandro; Clarke, Mike; Cardwell, Christopher R; Jones, Sophie; Maguire, Aideen; Ellis, Geraint; Murtagh, Brendan; Jurek-Loughrey, Anna; Bryan, Dominic; Barry, John; Hilton, Jeremy; Hafezi, Mehdi; Clewley, Natalie; Kee, Frank

---

VERSION 1 - REVIEW

**Reviewer**	**1**
**Name**	**McEachan, Rosemary**
**Affiliation**	**Bradford Teaching Hospitals NHS Foundation Trust, Bradford Institute for Health Research**
**Date**	**27-Feb-2025**
**COI**	**None**

Thanks for the opportunity to review this protocol. The proposed evaluation is comprehensive and will provide much needed evidence on the longer term health benefits of investing in green space and public realm. The strong focus on systems methods of evaluation is very welcome.

I have only a couple of minor comments to enable the reader to have a clearer understanding of certain areas of the research protocol.

Understanding systems:

Could the authors give a bit more detail on how some of these techniques are implemented. This would be really helpful for others who might want to implement this approach but who haven’t done it before.

For example, what format do the ‘collaborative conversations with key stakeholders’ take? Is this a formal or informal process, who are the stakeholders and how many?

In terms of the methods mentioned are these developed by the research team based on the conversations, or are developed in workshops?

Are these outputs developed iteratively?

Intercept surveys:

It would be helpful to have a bit more information on what these are and what they measure?

Patient and public involvement:

It is great to see strong public engagement embedded into this long term research evaluation – can the authors give us some examples of how community feedback has been used to shape the development of the research programme?

**Reviewer**	**2**
**Name**	**Crane, Melanie**
**Affiliation**	**University of Sydney School of Public Health**
**Date**	**02-Jun-2025**
**COI**	**None**

Thank you for the opportunity to review this protocol paper on the evaluation of a new urban greenway in Belfast. I am particularly encouraged to see your use of systems methods including CLDs to inform your evaluation – demonstrating the application of such methods in the development and co-design of evaluation frameworks in this area of research.

This is an ambitious evaluation but also one that is sorely needed. For such complex interventions as urban green developments mixed methods are necessary to inform the evaluation – particularly to evaluate system and population level impacts.

I concur this is one of the longest follow up evaluation of UGBS – and that is necessary to gather the type of outcomes intended here - having this wealth of linked administrative data will be very informative assuming there is sufficient time for lag effect.

The household survey recruitment method is one thing that I query – in terms of current landline phone availability. I am not familiar with Belfast phone use but many population surveys around the world have had to move away from CATI calling technology because of the less homes with landline phones –more households only having mobile access. This was a concern a decade ago leading to different methods but here you suggest you will choose the same approach as 2011. While consistency of methods for repeat analysis are justified - I just question whether the sample will be representative.

Sample sizes are appropriate for both he quantitative and qualitative data analyses and systems mapping. This data is appropriate to inform the economic evaluation.

I look forward to seeing the results of this study published.

VERSION 1 - AUTHOR RESPONSE

Reviewer 1:

4. Understanding systems:
Could the authors give a bit more detail on how some of these techniques are implemented. This would be really helpful for others who might want to implement this approach but who haven't done it before.
For example, what format do the 'collaborative conversations with key stakeholders' take? Is this a formal or informal process, who are the stakeholders and how many?
In terms of the methods mentioned are these developed by the research team based on the conversations, or are developed in workshops?
Are these outputs developed iteratively?

Author response: We have included additional detail on the methodological approaches as suggested.

"Initially, we will generate a shared understanding of the context and use of the CCG through formal collaborative conversations, surveys and workshops with approximately 30 stakeholders from local community groups, local authorities, government departments, urban designers, landscape architects, and local residents. Methods will include rich pictures (Checkland and Poulter, 2006; Checkland and Scholes, 1990; Flood & Carson, 1993; Wilson, 1990), multi-perspective diagrams (Dodd & Alston, 2009), context diagrams (Flood & Carson, 1993), root definitions (Checkland and Poulter, 2006; Checkland and Scholes, 1990; Wilson, 1990), stakeholder network analysis survey (Nigg et al, 2002; Provan et al, 2004), and the development of a Causal Loop Diagrams based on group model building methods (Stroh, 2015; Vandenbroeck et al., 2007). We will then model the contribution of stakeholders to the development, implementation and maintenance of the CCG with Viable Systems Model (Beer, 1981, 1985; Espejo and Reyes, 2011; Leonard, 2009), informed by Soft Systems Methodology Conceptual Models and the Causal Loop Diagrams. Critical dependencies will be determined by generating and analysing a Dependency Model (Slater, 2016). Measures of success can be elicited from the Soft Systems Methodology Conceptual Model activity analysis and analysis of the Causal Loop Diagrams (see Table 1 for descriptions of systems science methods). These proposed approaches can help decision-makers and planners identify the stakeholders required for each phase, their roles and responsibilities, and build capacity in local communities to develop amenities to meet their needs. The CCG will be modelled in two phases: 1) development and implementation; and, 2) management and maintenance. A "Reference Model" is then derived from integrating the data generated by the multiple systems methods used (Soft Systems Methodology Conceptual Model, Viable Systems Model, Causal Loop Diagram and Dependency Model). The outputs will be developed iteratively leading to a multi-perspective system model integrating the various methods."

5. Intercept surveys:
It would be helpful to have a bit more information on what these are and what they measure?

Author response: We have added further details re: the Intercept Survey methods and measures to the revised manuscript.

"The surveys will take place on four separate days at each of the four locations – 16 survey days in total. The days for each site (7am to 7pm) will be: term time weekday; term time weekend day; non term time weekday; non term time weekend day. Surveyors will count all cyclists, e-bikes, pedestrians, horse riders, roller skaters and wheelchair users passing the survey point, covering people travelling in all required directions – including those travelling on the neighbouring pavement, and on-road cycles. Other data collected will include route choice, distance travelled, perceptions of safety, physical activity and mental wellbeing."

6. Patient and public involvement:
It is great to see strong public engagement embedded into this long term research evaluation – can the authors give us some examples of how community feedback has been used to shape the development of the research programme?

Author response: Thank you for this suggestion. We have added the following text to illustrate the importance of the continued public engagement in this research in the Patient and Public Involvement section.

"Consultation and engagement with the local community has helped to shape the development of the research programme. For example, group model building workshops involving the local community led to inclusion of biodiversity, nature connectedness and sense of community pride measures being included in the wave 3 household survey and further explored in the qualitative workshops."

Reviewer 2:

7. The household survey recruitment method is one thing that I query – in terms of current landline phone availability. I am not familiar with Belfast phone use but many population surveys around the world have had to move away from CATI calling technology because of the less homes with landline phones –more households only having mobile access. This was a concern a decade ago leading to different methods but here you suggest you will choose the same approach as 2011. While consistency of methods for repeat analysis are justified - I just question whether the sample will be representative.

Author response: We apologise for the confusion with this paragraph. We have updated the text to change the term 'in-person calls' to 'in-person visits' to prevent confusion. Interviewers visit the household in-person. They do not conduct a phone call.

"Interviewers from Perceptive Insight Market Research Ltd will visit in-person to each household (including those who have not made contact) to recruit an adult (≥16 years of age) from each household to participate. Up to four in-person visits to the household will be made to achieve an interview".
